# A Novel Design of an Intelligent Drug Delivery System Based on Nanoantenna Particles

**DOI:** 10.1186/s11671-019-3102-z

**Published:** 2019-08-20

**Authors:** M. Abbas, A. Kessentini, H. Loukil, P. Muneer, V. P. Thafasal Ijyas, S. E. Bushara, M. Abdul Wase

**Affiliations:** 1 Electrical Engineering Department, College of Engineering, King Khalid University, P.O.Box 960, Abha, Asir 61421 Saudi Arabia; 2 Department of Mechanical Engineering, College of Engineering, King Khalid University, P.O.Box 960, Abha, Asir 61421 Saudi Arabia; 3 Department of Computers and Communications, College of Engineering, Delta University for Science and Technology, Talkha, Egypt; 4 Laboratory of Electromechanical Systems (LASEM), National Engineering School of Sfax, University of Sfax, Route de Soukra km 4, 3038 Sfax, Tunisia; 5 Electronics and Information Technology Laboratory, University of Sfax, National Engineering School of Sfax, Sfax, Tunisia

**Keywords:** Drug delivery, Nanoparticles, Fluid viscosity, Lymphatic fluid, Cancer cells

## Abstract

Compound nanoparticle drug delivery system plays an important role in the interaction with lymph nodes. There are three primary types of lymphocytes: B cells, T cells, and natural killer cells. When the cells of the immune system turn carcinogenic, they assault body cells. The lymph fluid plays an important role in attacking healthy cells of the body; hence, this paper aimed to design a drug delivery system, which can efficiently direct nanoparticles to target the infected cells, helping in high-speed elimination of such cells. The proposed design depends on the interaction between these molecules, and the intelligent nano-controller has the ability to guide the nanoparticles by anaerobic contact. The proposed design proved that the smaller the nanoparticle size and density, the less dynamic viscosity of the liquid would be, which would reflect its resistance to flow. In addition, it was concluded that hydrogen molecules play a significant role in reducing lymphatic fluid resistance due to their low density.

## Introduction

The current cancer treatment options include surgery, radiation, and chemotherapy. These treatment strategies also harm ordinary tissues and result in partial annihilation of malignant growth. Therefore, nanotechnology can overcome these shortcomings by specifically targeting harmful cells and neoplasm, directly resecting tumors, and increasing the effectiveness of radiation-based and other treatment modalities. This can significantly decrease the adverse effects of the treatment and increase the rate of survival. Nanotechnology is a promising tool for the treatment of malignant growth as it offers newer and better treatment modalities by using nanomaterial. Nanoparticles can specifically target many molecules differentially expressed on cancer cells. The generally vast airfoil region of nanoparticles can be functionalized with ligands such as small particles and deoxyribonucleic corrosive or ribonucleic corrosive chain peptide antibodies. The ligands are used as a drug and in theranostic applications. The physical properties of nanoparticles, such as vitality distraction and reradiation, can likewise be used to affect ailing tissue, such as in laser removal and hyperthermia applications [1].

**Fig. 1:**
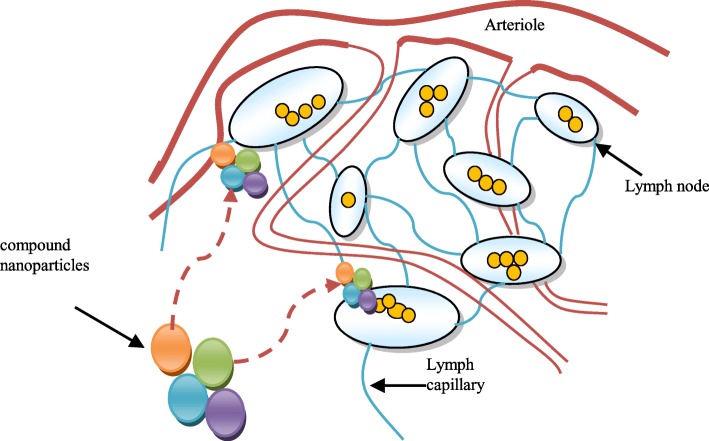
Compound nanoparticle drug delivery system and its interaction with lymph nodes

The innovative nanoparticle software program and active pharmaceutical element will also enable exploration of a wider repertoire of active ingredients. Therefore, the immunogenic cargo and surface coat are being investigated as both adjuvants to nanoparticle-mediated and traditional chemotherapy. This innovative strategy includes the design of nanoparticles as artificial antigen presenting on cells and in vivo depots of stimulatory factors that exert anti-tumor effects. The nanotechnology represents an active area of research with many applications. Nanoparticles have gained interest in medical-technology due to their tunable physicochemical characteristics such as thawing index point, hydrophilicity, electrical and thermal conduction, catalytic activity, light absorption, and scattering [2]. In principle, nanomaterials are described as materials with particles in the range of 1 to 100 nm. There are several pieces of legislation in the European Union and the USA with specific reference to medical research using nanomaterials. However, there is no internationally accepted definition of nanomaterials. Different organizations consider different concepts of nanomaterials [3]. One of the aims of the nanoparticle drug delivery system is to treat lymphatic fluid with cancer cells. A compound nanoparticle drug delivery system in interaction with lymph nodes is shown in Fig. 1.

The US Food and Drug Administration alludes to nanomaterials as materials with particles in the range of 1 to 100 with properties different from the bulk material [4, 5]. Nanofibers, nanoplates, nanowires, quantum dots, and other related materials have been characterized [6]. Solid lipid nanoparticles (SLNs) are a type of lipid nanoparticles (LN), which can be constructed by utilizing solid lipids [7]. Subsequent versions of SLNs have been developed such as nanostructured lipid carriers (NLCs), which represent the second era of LN [8]. Both SLN and NLC are built from solid lipids. The interior structure of SLN contains solid lipids, while NLC is developed utilizing a mixture of solid and liquid lipids, which produce precious stone cross section [9, 10]. These flaws have additionally been reported for SLNs in light of the fact that SLNs that contain many solid lipid segments can be used in medical applications [11, 12]. Polymeric nanoparticles (PN) can be built from natural polymers or inorganic materials, for example, silica [13]. The polymers or lipids shape the core of NPs, which improve stability and drug delivery and offer uniform shape and size [14]. PN can be described as nanocapsules or nanospheres. The nanocapsules contain oil in a vesicular structure along with a drug [15, 16], while nanospheres contain polymeric chains without oil [17, 18]. A drug is packed in PNs through blending with the polymer. The incorporation of the drug is ensured in the nanoparticles at the time of polymerization. PNs are loaded with a drug by dissolving, scattering, or artificially adsorbing it in the constituents of the polymer network [19, 20]. There are three types of lymphocytes: B cells, T cells, and natural killer cells. The B cells make antibodies that attack invading microorganisms, while they also attack the immune system when they become carcinogenic. Therefore, considering the important role of lymph fluid in autoimmunity, the objective of this paper was to design an intelligent drug delivery system based on nanoantenna particles. Thus, the system contains many nanoparticles in different quantities. The next section presents the design of an intelligent drug delivery system.

### Design of a Nano-Intelligent Drug Delivery System

**Fig. 2:**
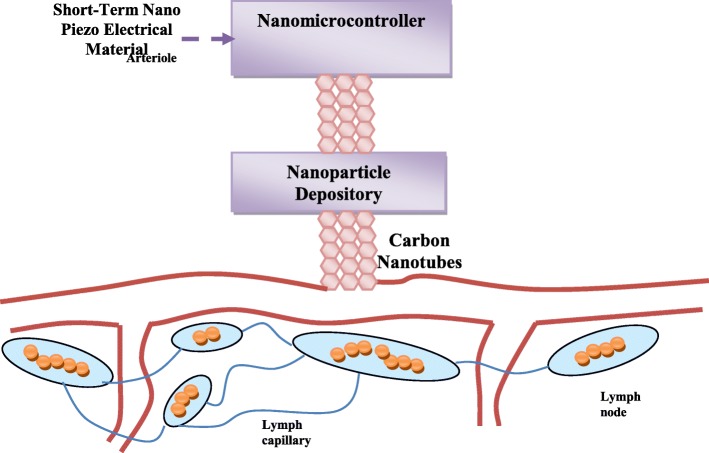
General structure showing the association of the proposed drug system with the infected cells

The proposed nano-intelligent drug delivery system contains a nano-controller operated by an electrical source of nanoparticles made of a nano-piezoelectric material. The complex repository of nanoparticles has a number of micro-repositories. Each small repository contains one type of nanoparticles. A nanoparticle molecule contains a nanoantenna designed to communicate with the nano-controller. The proposed nano-intelligent drug delivery system also contains carbon nanotubes for the rapid delivery of drugs to cancer cells. It can be associated with the infected cells as shown in Fig. 2. The system begins by sending nanoparticles to the cancer cells called “exploratory nanoparticles.” These molecules, by means of anaerobic communication, send the complete picture of their position inside the cells to the nano-controller. Based on the situation encountered by the exploratory nanoparticles, the nano-controller sends nanoparticles of different number, type, and density to the cancer cells, based on the information collected from the exploratory nanoparticles. These nanoparticles are called “fighting nanoparticles.”

**Fig. 3:**
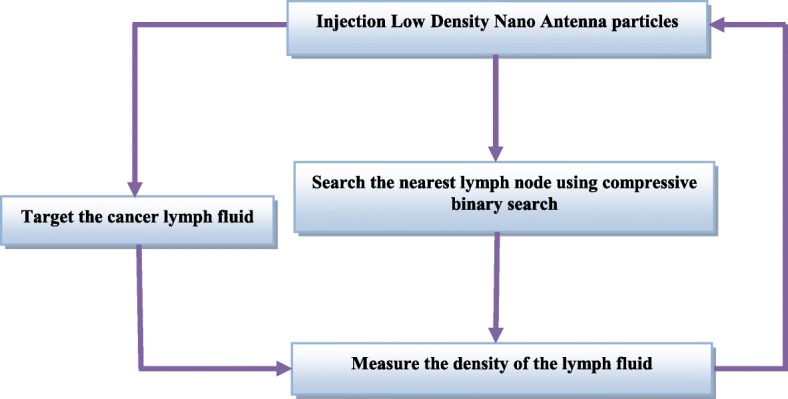
Sending process of the fighting nanoparticles to cancer cells

This is not a random process but controlled by the nano-controller considering several aspects and logarithms, which will ensure efficient and quick delivery of the nanoparticles. In order to accurately and quickly deliver the nanoparticles to the cancer cells, the compressive binary search algorithm will be employed [21]. Further, nanoparticles will be delivered in different densities so that the drug becomes more effective. These methodologies and their modus operandi by employing the nano-controller are illustrated in Fig. 3. The physical structure of the nano-controller is similar to that of the nanoparticles, but it is in the form of metal so that it can gain electrical energy for a short period while working. This metal contains a wireless antenna along with a small memory that contains the operating codes with a nanoparticle link between the nano-controller and the nanoparticle store. The nanoparticle repository contains several different types of nanoparticles. The opening and closing, as well as the duration of opening of the nano-gate, will be controlled to tailor the number of particles to be delivered.

### A Description of the Nature of the Nanoparticles Used in the Proposed Drug System

In the next section, the nature of the nanoparticles utilized in the proposed drug delivery system is discussed. In this work, low-density anaerobic nanoparticles were used as described in an earlier report [22].

#### Low-Density Nanoparticles

Consider the drug delivery process of compound nanoparticles for cancer as a penetrating process in lymph fluid, where a tumor is surrounded by the lymphatic fluid. The composition of the melanoma resembles the lymphatic fluid. The proposed analytical model is based on a nanotube system consisting of three different types of nanoparticles. The nanoparticles are placed in a high-density lymphatic fluid. We can define specific nanoparticles of solid A in the spherical polar coordinates as *A* = (ra, ϑ*a*, φa), where ra is the radial coordinate for the nanoparticle of solid A, ϑ*a* is the zenithal coordinate for the nanoparticle of solid A, and φa is the azimuthal coordinate for the nanoparticle of solid A. The corresponding coordinates of solid B are *B* = (rb, ϑb, φb), respectively, and the corresponding coordinates of solid N are *N* = (rn, ϑn, φn), respectively. Consider that there are two properties of the lymph node, viz., tender and swollen, which are affected by the cancer cells of Hodgkin’s lymphoma. The lymph node with tender property Tp can be described as Tp (*N*,*t*); this means that the value of Tp in association with the fluid of nanoparticles of solid N varies with the time. Now, let us consider that the total effect of the compound nanoparticles in the tender property is defined as:

1 $$ \mathrm{Tpt}=\mathrm{Tp}\ \left(A,t\right)+\mathrm{Tp}\ \left(B,t\right)+.\dots \dots \dots \dots +\mathrm{Tp}\ \left(N,t\right) $$

Consider the same case for the swollen property, which could be defined as:

2 $$ \mathrm{Tst}=\mathrm{Ts}\ \left(A,t\right)+\mathrm{Ts}\ \left(B,t\right)+.\dots \dots \dots \dots +\mathrm{Ts}\ \left(N,t\right) $$

From Eqs. 1 and 2, the rate of change of both properties with time can be determined as:

3 $$ \frac{\partial \left(\mathrm{Tp}\left(A,t\right)\right)}{\partial t}+\frac{\partial \left(\mathrm{Tp}\left(B,t\right)\right)}{\partial t}+\dots \frac{\partial \left(\mathrm{Tp}\left(N,t\right)\right)}{\partial t}=\frac{\mathrm{\partial Tp}(t)}{\mathrm{\partial t}} $$

4 $$ \frac{\partial \left(\mathrm{Ts}\left(A,t\right)\right)}{\partial t}+\frac{\partial \left(\mathrm{Ts}\left(B,t\right)\right)}{\partial t}+\dots \frac{\partial \left(\mathrm{Ts}\left(N,t\right)\right)}{\partial t}=\frac{\mathrm{\partial Ts}(t)}{\partial t} $$

The point in the lymph fluid that can be occupied by one nanoparticle of solid N is defined as:

5 $$ {\mathrm{Po}}_n=\mathrm{Po}{\left(\mathrm{po},t\right)}_n $$

Let us consider a nanoparticle of the solid N derivative of the tender lymph fluid is defined as $\frac{\partial {\left(\mathrm{Tp}\left(N,t\right)\right)}_{\mathrm{po}}}{\partial t}$, then the compound material derivative of the tender lymph fluid would be equal to:

6 $$ \frac{\partial {\left(\mathrm{Tp}\left(A,t\right)\right)}_{\mathrm{po}}}{\partial t}+\frac{\partial {\left(\mathrm{Tp}\left(B,t\right)\right)}_{\mathrm{po}}}{\partial t}+\dots \frac{\partial {\left(\mathrm{Tp}\left(N,t\right)\right)}_{\mathrm{po}}}{\partial t}=\frac{\mathrm{\partial Tp}{(t)}_{\mathrm{po}}}{\partial t} $$

7 $$ \frac{\partial {\left(\mathrm{Ts}\left(A,t\right)\right)}_{\mathrm{po}}}{\partial t}+\frac{\partial {\left(\mathrm{Ts}\left(B,t\right)\right)}_{\mathrm{po}}}{\partial t}+\dots \frac{\partial {\left(\mathrm{Ts}\left(N,t\right)\right)}_{\mathrm{po}}}{\partial t}=\frac{\mathrm{\partial Ts}{(t)}_{\mathrm{po}}}{\partial t} $$

The corresponding velocity components of the solid N are taken as (*v*_rn_, *v*_ϑn_, *v*_φn_). Then, the flow velocity of the particles of solid N is represented using Navier-Stokes equations at dynamic viscosity dν of the lymph fluid, and *p* is the pressure and *ρ* is the density of lymph fluid as follows:

8 $$ \frac{\partial {v}_{\mathrm{rn}}}{\partial t}+{v}_{\mathrm{rn}}\frac{\partial {v}_{\mathrm{rn}}}{\mathrm{\partial rn}}+\frac{v_{\upvartheta \mathrm{n}}}{\mathrm{rn}}\frac{\partial {v}_{\mathrm{rn}}}{\mathrm{\partial \upvartheta n}}+\frac{v_{\upvarphi \mathrm{n}}}{\mathrm{rn}\ \mathrm{sin}\upvartheta \mathrm{n}}\frac{\partial {v}_{\mathrm{rn}}}{\mathrm{\partial \upvarphi n}}-\frac{v_{\upvartheta \mathrm{n}}^2}{\mathrm{rn}}-\frac{v_{\upvarphi \mathrm{n}}^2}{\mathrm{rn}}+\frac{1}{\rho}\frac{\partial p}{\mathrm{\partial rn}}-\mathrm{d}\upnu \left[\frac{1}{{\mathrm{rn}}^2}\frac{\partial }{\mathrm{\partial rn}}\left({\mathrm{rn}}^2\frac{\partial {v}_{\mathrm{rn}}}{\mathrm{\partial rn}}\right)+\frac{1}{{\mathrm{rn}}^2\mathrm{sin}\upvartheta \mathrm{n}}\frac{\partial }{\mathrm{\partial \upvartheta n}}\left(\mathrm{sin}\upvartheta \mathrm{n}\frac{\partial {v}_{\mathrm{rn}}}{\mathrm{\partial \upvartheta n}}\right)+\frac{1}{{\mathrm{rn}}^2{\sin}^2\upvartheta \mathrm{n}}\frac{\partial^2{v}_{\mathrm{rn}}}{\partial {\upvarphi \mathrm{n}}^2}+-\frac{2{v}_{\mathrm{rn}}}{{\mathrm{rn}}^2}-\frac{2}{{\mathrm{rn}}^2\sin \upvartheta \mathrm{n}}\frac{\partial \left({v}_{\upvartheta \mathrm{n}}\mathrm{sin}\upvartheta \mathrm{n}\right)}{\mathrm{\partial \upvartheta n}}-\frac{2}{{\mathrm{rn}}^2\mathrm{sin}\upvartheta \mathrm{n}}\frac{\partial {v}_{\upvarphi \mathrm{n}}}{\partial_{\upvarphi \mathrm{n}}}\right]=0 $$

The dynamic viscosity dν of the lymph fluid is calculated as follows:

9 $$ \mathrm{d}\upnu =\frac{\left[\frac{\partial {v}_{\mathrm{rn}}}{\partial t}+{v}_{\mathrm{rn}}\frac{\partial {v}_{\mathrm{rn}}}{\mathrm{\partial rn}}+\frac{v_{\upvartheta \mathrm{n}}\partial {v}_{\mathrm{rn}}}{\mathrm{rn}\ \mathrm{\partial \upvartheta n}}+\frac{v_{\upvarphi \mathrm{n}}}{\mathrm{rn}\ \mathrm{sin}\upvartheta \mathrm{n}}\frac{\partial {v}_{\mathrm{rn}}}{\mathrm{\partial \upvarphi n}}-\frac{v_{\upvartheta \mathrm{n}}^2}{\mathrm{rn}}-\frac{v_{\upvarphi \mathrm{n}}^2}{\mathrm{rn}}+\frac{1\ \partial p}{\uprho \partial \mathrm{rn}}\right]}{\left[\frac{1}{{\mathrm{rn}}^2}\frac{\partial }{\mathrm{\partial rn}}\left({\mathrm{rn}}^2\frac{\partial {v}_{\mathrm{rn}}}{\mathrm{\partial rn}}\right)+\frac{1}{{\mathrm{rn}}^2\mathrm{sin}\upvartheta \mathrm{n}}\frac{\partial }{\mathrm{\partial \upvartheta n}}\left(\mathrm{sin}\upvartheta \mathrm{n}\frac{\partial {v}_{\mathrm{rn}}}{\mathrm{\partial \upvartheta n}}\right)+\frac{1}{{\mathrm{rn}}^2{\sin}^2\upvartheta \mathrm{n}}\frac{\partial^2{v}_{\mathrm{rn}}}{\partial {\upvarphi \mathrm{n}}^2}+-\frac{2{v}_{\mathrm{rn}}}{{\mathrm{rn}}^2}-\frac{2}{{\mathrm{rn}}^2\sin \upvartheta \mathrm{n}}\frac{\partial \left({v}_{\upvartheta \mathrm{n}}\mathrm{sin}\upvartheta \mathrm{n}\right)}{\mathrm{\partial \upvartheta n}}-\frac{2}{{\mathrm{rn}}^2\mathrm{sin}\upvartheta \mathrm{n}}\frac{\partial {v}_{\upvarphi \mathrm{n}}}{\partial_{\upvarphi \mathrm{n}}}\right]} $$

The Navier-Stokes equations of solids A and B could be represented as Eqs. 8 and 9. Thus, Eq. 9 could be represented as follows:

10 $$ \mathrm{d}\upnu =\frac{\left[\frac{\partial {v}_{\mathrm{rn}}}{\partial t}+{v}_{\mathrm{rn}}\frac{\partial {v}_{\mathrm{rn}}}{\mathrm{\partial rn}}+\frac{v_{\upvartheta \mathrm{n}}}{\mathrm{rn}}\frac{\partial {v}_{\mathrm{rn}}}{\mathrm{\partial \upvartheta n}}+\frac{v_{\upvarphi \mathrm{n}}}{\mathrm{rn}\ \mathrm{sin}\upvartheta \mathrm{n}}\frac{\partial {v}_{\mathrm{rn}}}{\mathrm{\partial \upvarphi n}}-\frac{v_{\upvartheta \mathrm{n}}^2}{\mathrm{rn}}-\frac{v_{\upvarphi \mathrm{n}}^2}{\mathrm{rn}}+\frac{1}{\rho}\frac{\partial p}{\mathrm{\partial rn}}\right]}{\left[\frac{1}{{\mathrm{rn}}^2}\frac{\partial }{\mathrm{\partial rn}}\left({\mathrm{rn}}^2\frac{\partial {v}_{\mathrm{rn}}}{\mathrm{\partial rn}}\right)+\frac{1}{{\mathrm{rn}}^2\mathrm{sin}\upvartheta \mathrm{n}}\frac{\partial }{\mathrm{\partial \upvartheta n}}\left(\mathrm{sin}\upvartheta \mathrm{n}\frac{\partial {\mathrm{v}}_{\mathrm{rn}}}{\mathrm{\partial \upvartheta n}}\right)+\frac{1}{{\mathrm{rn}}^2{\sin}^2\upvartheta \mathrm{n}}\frac{\partial^2{\mathrm{v}}_{\mathrm{rn}}}{\partial {\upvarphi \mathrm{n}}^2}-\frac{2{\mathrm{v}}_{\mathrm{rn}}}{{\mathrm{rn}}^2}-\frac{2}{{\mathrm{rn}}^2\sin \upvartheta \mathrm{n}}\frac{\partial \left({\mathrm{v}}_{\upvartheta \mathrm{n}}\mathrm{sin}\upvartheta \mathrm{n}\right)}{\mathrm{\partial \upvartheta n}}-\frac{2}{{\mathrm{rn}}^2\mathrm{sin}\upvartheta \mathrm{n}}\frac{\partial {\mathrm{v}}_{\upvarphi \mathrm{n}}}{\partial_{\upvarphi \mathrm{n}}}\right]}=\frac{\left[\frac{\partial {\mathrm{v}}_{\mathrm{ra}}}{\mathrm{\partial t}}+{\mathrm{v}}_{\mathrm{ra}}\frac{\partial {\mathrm{v}}_{\mathrm{ra}}}{\mathrm{\partial ra}}+\frac{{\mathrm{v}}_{\upvartheta \mathrm{a}}}{\mathrm{ra}}\frac{\partial {\mathrm{v}}_{\mathrm{ra}}}{\mathrm{\partial \upvartheta a}}+\frac{{\mathrm{v}}_{\upvarphi \mathrm{a}}}{\mathrm{ra}\ \mathrm{sin}\upvartheta \mathrm{a}}\frac{\partial {\mathrm{v}}_{\mathrm{ra}}}{\mathrm{\partial \upvarphi a}}-\frac{{\mathrm{v}}_{\upvartheta \mathrm{a}}^2}{\mathrm{ra}}-\frac{{\mathrm{v}}_{\upvarphi \mathrm{a}}^2}{\mathrm{ra}}+\frac{1}{\uprho}\frac{\mathrm{\partial p}}{\mathrm{\partial ra}}\right]}{\left[\frac{1}{{\mathrm{ra}}^2}\frac{\partial }{\mathrm{\partial ra}}\left({\mathrm{ra}}^2\frac{\partial {v}_{\mathrm{ra}}}{\mathrm{\partial ra}}\right)+\frac{1}{{\mathrm{ra}}^2\mathrm{sin}\upvartheta \mathrm{a}}\frac{\partial }{\mathrm{\partial \upvartheta a}}\left(\mathrm{sin}\upvartheta \mathrm{a}\frac{\partial {v}_{\mathrm{ra}}}{\mathrm{\partial \upvartheta a}}\right)+\frac{1}{{\mathrm{ra}}^2{\sin}^2\upvartheta \mathrm{a}}\frac{\partial^2{v}_{\mathrm{ra}}}{\partial {\upvarphi \mathrm{a}}^2}-\frac{2{v}_{\mathrm{ra}}}{{\mathrm{ra}}^2}-\frac{2}{{\mathrm{ra}}^2\sin \upvartheta \mathrm{a}}\frac{\partial \left({v}_{\upvartheta \mathrm{a}}\mathrm{sin}\upvartheta \mathrm{a}\right)}{\mathrm{\partial \upvartheta a}}-\frac{2}{{\mathrm{ra}}^2\mathrm{sin}\upvartheta \mathrm{a}}\frac{\partial {v}_{\upvarphi \mathrm{a}}}{\partial_{\upvarphi \mathrm{a}}}\right]}=\frac{\left[\frac{\partial {v}_{\mathrm{rb}}}{\partial t}+{v}_{\mathrm{rb}}\frac{\partial {v}_{\mathrm{rb}}}{\mathrm{\partial rb}}+\frac{v_{\upvartheta \mathrm{b}}}{\mathrm{rb}}\frac{\partial {v}_{\mathrm{rb}}}{\mathrm{\partial \upvartheta b}}+\frac{v_{\upvarphi \mathrm{b}}}{\mathrm{rb}\ \mathrm{sin}\upvartheta \mathrm{b}}\frac{\partial {v}_{\mathrm{rb}}}{\mathrm{\partial \upvarphi b}}-\frac{v_{\upvartheta \mathrm{b}}^2}{\mathrm{rb}}-\frac{v_{\upvarphi \mathrm{b}}^2}{\mathrm{rb}}+\frac{1}{\rho}\frac{\partial p}{\mathrm{\partial rb}}\right]}{\left[\frac{1}{{\mathrm{rb}}^2}\frac{\partial }{\mathrm{\partial rb}}\left({\mathrm{rb}}^2\frac{\partial {v}_{\mathrm{rb}}}{\mathrm{\partial rb}}\right)+\frac{1}{{\mathrm{rb}}^2\mathrm{sin}\upvartheta \mathrm{b}}\frac{\partial }{\mathrm{\partial \upvartheta b}}\left(\mathrm{sin}\upvartheta \mathrm{b}\frac{\partial {v}_{\mathrm{rb}}}{\mathrm{\partial \upvartheta b}}\right)+\frac{1}{{\mathrm{rb}}^2{\sin}^2\upvartheta \mathrm{b}}\frac{\partial^2{v}_{\mathrm{rb}}}{\partial {\upvarphi \mathrm{b}}^2}-\frac{2{v}_{\mathrm{rb}}}{{\mathrm{rb}}^2}-\frac{2}{{\mathrm{rb}}^2\sin \upvartheta \mathrm{b}}\frac{\partial \left({v}_{\upvartheta \mathrm{b}}\mathrm{sin}\upvartheta \mathrm{b}\right)}{\mathrm{\partial \upvartheta b}}-\frac{2}{{\mathrm{rb}}^2\mathrm{sin}\upvartheta \mathrm{b}}\frac{\partial {v}_{\upvarphi \mathrm{b}}}{\partial_{\upvarphi \mathrm{b}}}\right]} $$

The particles are in nano-dimensions; thus, their radii would be very small, and for simplicity, Eq. 10 is represented as follows:

11 $$ \mathrm{d}\upnu =\left[\frac{v_{\upvartheta \mathrm{n}}}{\mathrm{rn}}\frac{\partial {v}_{\mathrm{rn}}}{\mathrm{\partial \upvartheta n}}+\frac{v_{\upvarphi \mathrm{n}}}{\mathrm{rn}\ \mathrm{sin}\upvartheta \mathrm{n}}\frac{\partial {v}_{\mathrm{rn}}}{\mathrm{\partial \upvarphi n}}-\frac{v_{\upvartheta \mathrm{n}}^2}{\mathrm{rn}}-\frac{v_{\upvarphi \mathrm{n}}^2}{\mathrm{rn}}+\frac{1}{\rho}\frac{\partial p}{\mathrm{\partial rn}}\right]/\left[\frac{1}{{\mathrm{rn}}^2}\frac{\partial }{\mathrm{\partial rn}}\left({\mathrm{rn}}^2\frac{\partial {v}_{\mathrm{rn}}}{\mathrm{\partial rn}}\right)+\frac{1}{{\mathrm{rn}}^2\mathrm{sin}\upvartheta \mathrm{n}}\frac{\partial }{\mathrm{\partial \upvartheta n}}\left(\mathrm{sin}\upvartheta \mathrm{n}\frac{\partial {\mathrm{v}}_{\mathrm{rn}}}{\mathrm{\partial \upvartheta n}}\right)+\frac{1}{{\mathrm{rn}}^2{\sin}^2\upvartheta \mathrm{n}}\frac{\partial^2{\mathrm{v}}_{\mathrm{rn}}}{\partial {\upvarphi \mathrm{n}}^2}-\frac{2{\mathrm{v}}_{\mathrm{rn}}}{{\mathrm{rn}}^2}-\frac{2}{{\mathrm{rn}}^2\sin \upvartheta \mathrm{n}}\frac{\partial \left({\mathrm{v}}_{\upvartheta \mathrm{n}}\mathrm{sin}\upvartheta \mathrm{n}\right)}{\mathrm{\partial \upvartheta n}}-\frac{2}{{\mathrm{rn}}^2\mathrm{sin}\upvartheta \mathrm{n}}\frac{\partial {\mathrm{v}}_{\upvarphi \mathrm{n}}}{\partial_{\upvarphi \mathrm{n}}}\right]=\left[\frac{{\mathrm{v}}_{\upvartheta \mathrm{a}}}{\mathrm{ra}}\frac{\partial {\mathrm{v}}_{\mathrm{ra}}}{\mathrm{\partial \upvartheta a}}+\frac{{\mathrm{v}}_{\upvarphi \mathrm{a}}}{\mathrm{ra}\ \mathrm{sin}\upvartheta \mathrm{a}}\frac{\partial {\mathrm{v}}_{\mathrm{ra}}}{\mathrm{\partial \upvarphi a}}-\frac{{\mathrm{v}}_{\upvartheta \mathrm{a}}^2}{\mathrm{ra}}-\frac{{\mathrm{v}}_{\upvarphi \mathrm{a}}^2}{\mathrm{ra}}+\frac{1}{\uprho}\frac{\mathrm{\partial p}}{\mathrm{\partial ra}}\right]/\left[\frac{1}{{\mathrm{ra}}^2}\frac{\partial }{\mathrm{\partial ra}}\left({\mathrm{ra}}^2\frac{\partial {\mathrm{v}}_{\mathrm{ra}}}{\mathrm{\partial ra}}\right)+\frac{1}{{\mathrm{ra}}^2\mathrm{sin}\upvartheta \mathrm{a}}\frac{\partial }{\mathrm{\partial \upvartheta a}}\left(\mathrm{sin}\upvartheta \mathrm{a}\frac{\partial {\mathrm{v}}_{\mathrm{ra}}}{\mathrm{\partial \upvartheta a}}\right)+\frac{1}{{\mathrm{ra}}^2{\sin}^2\upvartheta \mathrm{a}}\frac{\partial^2{\mathrm{v}}_{\mathrm{ra}}}{\partial {\upvarphi \mathrm{a}}^2}-\frac{2{\mathrm{v}}_{\mathrm{ra}}}{{\mathrm{ra}}^2}-\frac{2}{{\mathrm{ra}}^2\sin \upvartheta \mathrm{a}}\frac{\partial \left({\mathrm{v}}_{\upvartheta \mathrm{a}}\mathrm{sin}\upvartheta \mathrm{a}\right)}{\mathrm{\partial \upvartheta a}}-\frac{2}{{\mathrm{ra}}^2\mathrm{sin}\upvartheta \mathrm{a}}\frac{\partial {v}_{\upvarphi \mathrm{a}}}{\partial_{\upvarphi \mathrm{a}}}\right]=\left[\frac{v_{\upvartheta \mathrm{b}}}{\mathrm{rb}}\frac{\partial {v}_{\mathrm{rb}}}{\mathrm{\partial \upvartheta b}}+\frac{v_{\upvarphi \mathrm{b}}}{\mathrm{rb}\ \mathrm{sin}\upvartheta \mathrm{b}}\frac{\partial {v}_{\mathrm{rb}}}{\mathrm{\partial \upvarphi b}}-\frac{v_{\upvartheta \mathrm{b}}^2}{\mathrm{rb}}-\frac{v_{\upvarphi \mathrm{b}}^2}{\mathrm{rb}}+\frac{1}{\rho}\frac{\partial p}{\mathrm{\partial rb}}\right]/\left[\frac{1}{{\mathrm{rb}}^2}\frac{\partial }{\mathrm{\partial rb}}\left({\mathrm{rb}}^2\frac{\partial {v}_{\mathrm{rb}}}{\mathrm{\partial rb}}\right)+\frac{1}{{\mathrm{rb}}^2\mathrm{sin}\upvartheta \mathrm{b}}\frac{\partial }{\mathrm{\partial \upvartheta b}}\left(\mathrm{sin}\upvartheta \mathrm{b}\frac{\partial {v}_{\mathrm{rb}}}{\mathrm{\partial \upvartheta b}}\right)+\frac{1}{{\mathrm{rb}}^2{\sin}^2\upvartheta \mathrm{b}}\frac{\partial^2{v}_{\mathrm{rb}}}{\partial {\upvarphi \mathrm{b}}^2}-\frac{2{v}_{\mathrm{rb}}}{{\mathrm{rb}}^2}-\frac{2}{{\mathrm{rb}}^2\sin \upvartheta \mathrm{b}}\frac{\partial \left({v}_{\upvartheta \mathrm{b}}\mathrm{sin}\upvartheta \mathrm{b}\right)}{\mathrm{\partial \upvartheta b}}-\frac{2}{{\mathrm{rb}}^2\mathrm{sin}\upvartheta \mathrm{b}}\frac{\partial {v}_{\upvarphi \mathrm{b}}}{\partial_{\upvarphi \mathrm{b}}}\right] $$

Equation 11 could be represented as follows:

12 $$ \mathrm{d}\upnu =\mathrm{rn}\left[{v}_{\upvartheta \mathrm{n}}\frac{\partial {v}_{\mathrm{rn}}}{\mathrm{\partial \upvartheta n}}+\frac{v_{\upvarphi \mathrm{n}}}{\ \mathrm{sin}\upvartheta \mathrm{n}}\frac{\partial {v}_{\mathrm{rn}}}{\mathrm{\partial \upvarphi n}}-{v}_{\upvartheta \mathrm{n}}^2-{v}_{\upvarphi \mathrm{n}}^2+\frac{\mathrm{rn}}{\rho}\frac{\partial p}{\mathrm{\partial rn}}\right]/\left[\frac{\partial }{\mathrm{\partial rn}}\left({\mathrm{rn}}^2\frac{\partial {v}_{\mathrm{rn}}}{\mathrm{\partial rn}}\right)+\frac{1}{\mathrm{sin}\upvartheta \mathrm{n}}\frac{\partial }{\mathrm{\partial \upvartheta n}}\left(\mathrm{sin}\upvartheta \mathrm{n}\frac{\partial {v}_{\mathrm{rn}}}{\mathrm{\partial \upvartheta n}}\right)+\frac{1}{\sin^2\upvartheta \mathrm{n}}\frac{\partial^2{v}_{\mathrm{rn}}}{\partial {\upvarphi \mathrm{n}}^2}-2{v}_{\mathrm{rn}}-\frac{2}{\sin \upvartheta \mathrm{n}}\frac{\partial \left({v}_{\upvartheta \mathrm{n}}\mathrm{sin}\upvartheta \mathrm{n}\right)}{\mathrm{\partial \upvartheta n}}-\frac{2}{\mathrm{sin}\upvartheta \mathrm{n}}\frac{\partial {v}_{\upvarphi \mathrm{n}}}{\partial_{\upvarphi \mathrm{n}}}\right]=\mathrm{ra}\left[{v}_{\upvartheta \mathrm{a}}\frac{\partial {v}_{\mathrm{ra}}}{\mathrm{\partial \upvartheta a}}+\frac{v_{\upvarphi \mathrm{a}}}{\ \mathrm{sin}\upvartheta \mathrm{a}}\frac{\partial {v}_{\mathrm{ra}}}{\mathrm{\partial \upvarphi a}}-{v}_{\upvartheta \mathrm{a}}^2-{v}_{\upvarphi \mathrm{a}}^2+\frac{\mathrm{ra}}{\rho}\frac{\partial p}{\mathrm{\partial ra}}\right]/\left[\frac{\partial }{\mathrm{\partial ra}}\left({\mathrm{ra}}^2\frac{\partial {v}_{\mathrm{ra}}}{\mathrm{\partial ra}}\right)+\frac{1}{\mathrm{sin}\upvartheta \mathrm{a}}\frac{\partial }{\mathrm{\partial \upvartheta a}}\left(\mathrm{sin}\upvartheta \mathrm{a}\frac{\partial {v}_{\mathrm{ra}}}{\mathrm{\partial \upvartheta a}}\right)+\frac{1}{\sin^2\upvartheta \mathrm{a}}\frac{\partial^2{v}_{\mathrm{ra}}}{\partial {\upvarphi \mathrm{a}}^2}-2{v}_{\mathrm{ra}}-\frac{2}{\sin \upvartheta \mathrm{a}}\frac{\partial \left({v}_{\upvartheta \mathrm{a}}\mathrm{sin}\upvartheta \mathrm{a}\right)}{\mathrm{\partial \upvartheta a}}-\frac{2}{\mathrm{sin}\upvartheta \mathrm{a}}\frac{\partial {v}_{\upvarphi \mathrm{a}}}{\partial_{\upvarphi \mathrm{a}}}\right]=\mathrm{rb}\left[{v}_{\upvartheta \mathrm{b}}\frac{\partial {v}_{\mathrm{rb}}}{\mathrm{\partial \upvartheta b}}+\frac{v_{\upvarphi \mathrm{b}}}{\ \mathrm{sin}\upvartheta \mathrm{b}}\frac{\partial {v}_{\mathrm{rb}}}{\mathrm{\partial \upvarphi b}}-{v}_{\upvartheta \mathrm{b}}^2-{v}_{\upvarphi \mathrm{b}}^2+\frac{\mathrm{rb}}{\rho}\frac{\partial p}{\mathrm{\partial rb}}\right]/\left[\frac{\partial }{\mathrm{\partial rb}}\left({\mathrm{rb}}^2\frac{\partial {v}_{\mathrm{rb}}}{\mathrm{\partial rb}}\right)+\frac{1}{\mathrm{sin}\upvartheta \mathrm{b}}\frac{\partial }{\mathrm{\partial \upvartheta b}}\left(\mathrm{sin}\upvartheta \mathrm{b}\frac{\partial {v}_{\mathrm{rb}}}{\mathrm{\partial \upvartheta b}}\right)+\frac{1}{\sin^2\upvartheta \mathrm{b}}\frac{\partial^2{v}_{\mathrm{rb}}}{\partial {\upvarphi \mathrm{b}}^2}-2{v}_{\mathrm{rb}}-\frac{2}{\sin \upvartheta \mathrm{b}}\frac{\partial \left({v}_{\upvartheta \mathrm{b}}\mathrm{sin}\upvartheta \mathrm{b}\right)}{\mathrm{\partial \upvartheta b}}-\frac{2}{\mathrm{sin}\upvartheta \mathrm{b}}\frac{\partial {v}_{\upvarphi \mathrm{b}}}{\partial_{\upvarphi \mathrm{b}}}\right] $$

There is a direct relationship between the radii of the nanoparticles and the lymph viscosity due to cancer. If lymph becomes too static and viscous, it is unable to carry out its function properly, which is circulating and cleaning up toxins and helping to fight cancer. If nanoparticle size is smaller, the lymphatic cancer cells are easy to kill. In order to describe the transport of the total quantity of the compound nanoparticles, we use the continuity equation and assume the three nanoparticles of solids A, B, and N as follows:

13 $$ \frac{1}{{\mathrm{ra}}^2}\frac{\partial }{\mathrm{\partial ra}}\left({\mathrm{ra}}^2{v}_{\mathrm{ra}}\right)+\frac{1}{\mathrm{ra}\ \mathrm{sin}\upvartheta \mathrm{a}}\frac{\partial }{\mathrm{\partial \upvartheta a}}\left(\sin {\upvartheta \mathrm{v}}_{\upvartheta \mathrm{a}}\right)+\frac{1}{\mathrm{ra}\ \mathrm{sin}\upvartheta \mathrm{a}}\frac{\partial {v}_{\upvarphi \mathrm{a}}}{\mathrm{\partial \upvarphi a}}+\frac{1}{{\mathrm{rb}}^2}\frac{\partial }{\mathrm{\partial rb}}\left({\mathrm{rb}}^2{v}_{\mathrm{rb}}\right)+\frac{1}{\mathrm{rb}\ \mathrm{sin}\upvartheta \mathrm{b}}\frac{\partial }{\mathrm{\partial \upvartheta b}}\left(\sin {\upvartheta v}_{\upvartheta \mathrm{b}}\right)+\frac{1}{\mathrm{rb}\ \mathrm{sin}\upvartheta \mathrm{b}}\frac{\partial {v}_{\upvarphi \mathrm{b}}}{\mathrm{\partial \upvarphi b}}+\frac{1}{{\mathrm{rn}}^2}\frac{\partial }{\mathrm{\partial rn}}\left({\mathrm{rn}}^2{v}_{\mathrm{rn}}\right)+\frac{1}{\mathrm{rn}\ \mathrm{sin}\upvartheta \mathrm{n}}\frac{\partial }{\mathrm{\partial \upvartheta n}}\left(\sin {\upvartheta v}_{\upvartheta \mathrm{n}}\right)+\frac{1}{\mathrm{rn}\ \mathrm{sin}\upvartheta \mathrm{n}}\frac{\partial {v}_{\upvarphi \mathrm{n}}}{\mathrm{\partial \upvarphi n}}=0 $$

The dynamic fluid viscosity could be determined from the following equation [23]:

14 $$ \mathrm{Vs}=\frac{2}{9}\frac{r^2g\ \left(\uprho \mathrm{p}-\uprho \mathrm{f}\right)}{\mathrm{dv}} $$

where Vs is the particles’ settling velocity (m/s), *r* is the Stokes radius of the particle (m), *g* is the gravitational acceleration (m/s^2^), ρp is the density of the particles (kg/m^3^), ρf is the density of the fluid (kg/m^3^), and dv is the (dynamic) fluid viscosity (Pa·s). The lymph fluid is slightly heavier than water (lymph density = 1019 kg/m^3^, water density = 998.28 kg/m^3^ at 20 °C). As a reference value, we consider the dynamic viscosity of the water to be 1.002 × 10^–3^ kg m^–1^ s^–1^).

Dynamic viscosity is the measurement of the fluid’s internal resistance to flow, while kinematic viscosity refers to the ratio of dynamic viscosity to density. The effect of all the nanoparticles on the fluid viscosity is represented as follows:

15 $$ \mathrm{dv}=\frac{2\mathrm{g}}{9}\left[\frac{{\mathrm{ra}}^2\left(\uprho \mathrm{a}-\uprho \mathrm{f}\right)}{\mathrm{vsa}}+\frac{{\mathrm{rb}}^2\left(\uprho \mathrm{b}-\uprho \mathrm{f}\right)}{\mathrm{vsb}}+\frac{{\mathrm{rn}}^2\left(\uprho \mathrm{n}-\uprho \mathrm{f}\right)}{\mathrm{vn}}\right] $$

By comparing Eq. 12 and Eq. 15, the following equation could be emerged:

16 $$ \left|\frac{2\mathrm{g}}{9}\left[\frac{{\mathrm{ra}}^2\left(\uprho \mathrm{a}-\uprho \mathrm{f}\right)}{\mathrm{vsa}}+\frac{{\mathrm{rb}}^2\left(\uprho \mathrm{b}-\uprho \mathrm{f}\right)}{\mathrm{vsb}}+\frac{{\mathrm{rn}}^2\left(\uprho \mathrm{n}-\uprho \mathrm{f}\right)}{\mathrm{vn}}\right]\right|=\mathrm{rn}\left[{v}_{\upvartheta \mathrm{n}}\frac{\partial {v}_{\mathrm{rn}}}{\mathrm{\partial \upvartheta n}}+\frac{v_{\upvarphi \mathrm{n}}}{\ \mathrm{sin}\upvartheta \mathrm{n}}\frac{\partial {v}_{\mathrm{rn}}}{\mathrm{\partial \upvarphi n}}-{v}_{\upvartheta \mathrm{n}}^2-{v}_{\upvarphi \mathrm{n}}^2+\frac{\mathrm{rn}}{\uprho \mathrm{f}}\frac{\mathrm{\partial p}}{\mathrm{\partial rn}}\right]/\left[\frac{\partial }{\mathrm{\partial rn}}\left({\mathrm{rn}}^2\frac{\partial {v}_{\mathrm{rn}}}{\mathrm{\partial rn}}\right)+\frac{1}{\mathrm{sin}\upvartheta \mathrm{n}}\frac{\partial }{\mathrm{\partial \upvartheta n}}\left(\mathrm{sin}\upvartheta \mathrm{n}\frac{\partial {v}_{\mathrm{rn}}}{\mathrm{\partial \upvartheta n}}\right)+\frac{1}{\sin^2\upvartheta \mathrm{n}}\frac{\partial^2{v}_{\mathrm{rn}}}{\partial {\upvarphi \mathrm{n}}^2}-2{v}_{\mathrm{rn}}-\frac{2}{\sin \upvartheta \mathrm{n}}\frac{\partial \left({v}_{\upvartheta \mathrm{n}}\mathrm{sin}\upvartheta \mathrm{n}\right)}{\mathrm{\partial \upvartheta n}}-\frac{2}{\mathrm{sin}\upvartheta \mathrm{n}}\frac{\partial {v}_{\upvarphi \mathrm{n}}}{\partial_{\upvarphi \mathrm{n}}}\right]=\mathrm{ra}\left[{v}_{\upvartheta \mathrm{a}}\frac{\partial {v}_{\mathrm{ra}}}{\mathrm{\partial \upvartheta a}}+\frac{v_{\upvarphi \mathrm{a}}}{\ \mathrm{sin}\upvartheta \mathrm{a}}\frac{\partial {v}_{\mathrm{ra}}}{\mathrm{\partial \upvarphi a}}-{v}_{\upvartheta \mathrm{a}}^2-{v}_{\upvarphi \mathrm{a}}^2+\frac{\mathrm{ra}}{\uprho \mathrm{f}}\frac{\partial p}{\mathrm{\partial ra}}\right]/\left[\frac{\partial }{\mathrm{\partial ra}}\left({\mathrm{ra}}^2\frac{\partial {v}_{\mathrm{ra}}}{\mathrm{\partial ra}}\right)+\frac{1}{\mathrm{sin}\upvartheta \mathrm{a}}\frac{\partial }{\mathrm{\partial \upvartheta a}}\left(\mathrm{sin}\upvartheta \mathrm{a}\frac{\partial {v}_{\mathrm{ra}}}{\mathrm{\partial \upvartheta a}}\right)+\frac{1}{\sin^2\upvartheta \mathrm{a}}\frac{\partial^2{v}_{\mathrm{ra}}}{\partial {\upvarphi \mathrm{a}}^2}-2{v}_{\mathrm{ra}}-\frac{2}{\sin \upvartheta \mathrm{a}}\frac{\partial \left({v}_{\upvartheta \mathrm{a}}\mathrm{sin}\upvartheta \mathrm{a}\right)}{\mathrm{\partial \upvartheta a}}-\frac{2}{\mathrm{sin}\upvartheta \mathrm{a}}\frac{\partial {v}_{\upvarphi \mathrm{a}}}{\partial_{\upvarphi \mathrm{a}}}\right]=\mathrm{rb}\left[{v}_{\upvartheta \mathrm{b}}\frac{\partial {v}_{\mathrm{rb}}}{\mathrm{\partial \upvartheta b}}+\frac{v_{\upvarphi \mathrm{b}}}{\ \mathrm{sin}\upvartheta \mathrm{b}}\frac{\partial {v}_{\mathrm{rb}}}{\mathrm{\partial \upvarphi b}}-{v}_{\upvartheta \mathrm{b}}^2-{v}_{\upvarphi \mathrm{b}}^2+\frac{\mathrm{rb}}{\uprho \mathrm{f}}\frac{\partial p}{\mathrm{\partial rb}}\right]/\left[\frac{\partial }{\mathrm{\partial rb}}\left({\mathrm{rb}}^2\frac{\partial {v}_{\mathrm{rb}}}{\mathrm{\partial rb}}\right)+\frac{1}{\mathrm{sin}\upvartheta \mathrm{b}}\frac{\partial }{\mathrm{\partial \upvartheta b}}\left(\mathrm{sin}\upvartheta \mathrm{b}\frac{\partial {v}_{\mathrm{rb}}}{\mathrm{\partial \upvartheta b}}\right)+\frac{1}{\sin^2\upvartheta \mathrm{b}}\frac{\partial^2{v}_{\mathrm{rb}}}{\partial {\upvarphi \mathrm{b}}^2}-2{v}_{\mathrm{rb}}-\frac{2}{\sin \upvartheta \mathrm{b}}\frac{\partial \left({v}_{\upvartheta \mathrm{b}}\mathrm{sin}\upvartheta \mathrm{b}\right)}{\mathrm{\partial \upvartheta b}}-\frac{2}{\mathrm{sin}\upvartheta \mathrm{b}}\frac{\partial {v}_{\upvarphi \mathrm{b}}}{\partial_{\upvarphi \mathrm{b}}}\right] $$

Equation 16 depicts the relationship between the density of the lymph fluid, the density of the nanoparticles of the compound drug system, and the radius of the nanoparticles. There is a positive correlation between the density of the lymph fluid and the density of the nanoparticles. The smaller the density and radius of nanoparticles, the lesser the density of the fluid will be. As established earlier, the decrease in the density of the lymph fluid leads to its inability to reproduce and reduce the ferocity of the disease. It can, therefore, be concluded that the tumor can be cured by minimizing the size of nanoparticles. These particles can reach in the range of up to 0.1 nm (i.e., Angstrom or picometer range). The particles of this size can act as the nucleus of drug delivery in this drug system. Equation 16 shows that radii of the nanoparticles in the proposed drug system are related to the effectiveness of the delivery system. Much lower sized nanoparticles can reduce the density of lymph fluid and the spread of the disease.

#### Nanoparticles with Nanoantennas

This study used the nanoparticle described in an earlier report [22] as an emissary with a nano-microcontroller. In the system, the proposed transmission distance is very small and compatible with the composition of nanoparticles. Thus, the middle gap can be neglected in mid-distance and is symbolized by *C*_d_. Further, *R*_a_ and *X*_a_ are the real part and the imaginary part of the anaerobic impedance. After neglecting the load of the intercellular space between the nanoparticles and the nano-microcontroller, *R*_a_ and *X*_a_ can be calculated as follows [22]:

17 $$ {R}_a=\frac{r_{a0}}{1+{C}_d{w}_a\left(2{x}_{a0}+{C}_d\left({r_{a0}}^2+{x_{a0}}^2\right){w}_a\right)} $$

18 $$ {X}_a=\frac{x_0-{C}_d\left({r_{a0}}^2+{x_{a0}}^2\right){w}_a}{1+{C}_d{w}_a\left(2{x}_{a0}+{C}_d\left({r_{a0}}^2+{x_{a0}}^2\right){w}_a\right)} $$

Thus, the load resistance value of nanotubes can be predicted as in the following equation:

19 $$ {r}_l=\frac{g^2R}{g^2-2 gSX{\varepsilon}_L\omega +{S}^2\left({R}^2+{X}^2\right){\varepsilon_L}^2{\omega}^2} $$

where *ε*_*L*_ is the permittivity of the loading material, *g* is the size of the gap, and *S* is the effective cross-section area of the gap. In order to simplify the equation, the value of *g*^2^ can be neglected as it is too low and the final equation can be rewritten as follows:

20 $$ {r}_l=\frac{g^2R}{-2 gSX{\varepsilon}_L\omega +{S}^2\left({R}^2+{X}^2\right){\varepsilon_L}^2{\omega}^2} $$

Then,

21 $$ {r}_l=\frac{g^2R}{S{\varepsilon}_L\omega \left(-2 gX+S\left({R}^2+{X}^2\right){\varepsilon}_L\omega \right)} $$

The optical nano-photo concept can be used as an effective tool for interpreting and predicting these effects to design and improve nanoscale parameters and increase the nano-sensitivity to serve better as a single molecular sensor. Nanoantenna may provide optimal performance in terms of sensitivity, efficiency, and bandwidth in the process. The next section presents the concept of searching the cancerous lymph nodes using compressive binary search algorithm.

## Searching for the Target Lymphatic Nodes Using Compressive Binary Search

In order for nanoparticles to reach the cancerous cells in a fast and efficient manner, we applied compressive binary search by the nano-microcontroller. The guided nanoparticles follow a specific path to quickly reach the target. This movement is based on the information obtained from the “exploratory nanoparticles.” Assume that the target lymph node, Tf, has exactly one nonzero entry, where the location of the lymph node is unknown. The algorithm divides mt measurements into a total of St stages, where St refers to the stages of the lymph nodes. The measurements are more than one for all the stages of the lymph nodes, which is necessary for the algorithm to be executed until completion. Based on this measurement, the algorithm decides between going left or right, until the nanoparticles reach the target, the cancerous lymph node.

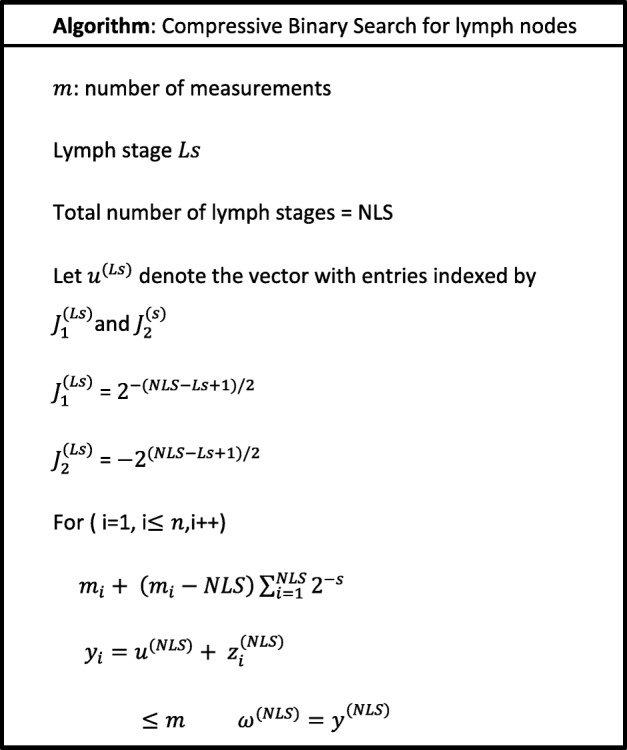



## Results and Discussion

**Fig. 4:**
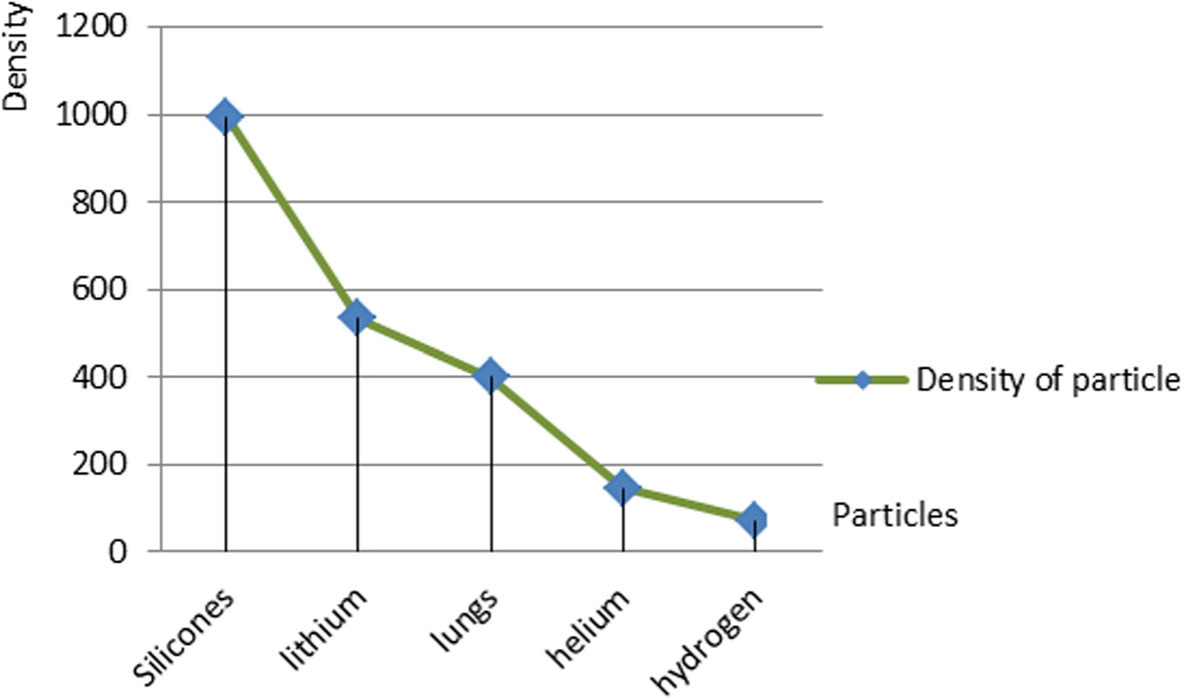
Density of nanoparticles for the five selected materials

**Fig. 5:**
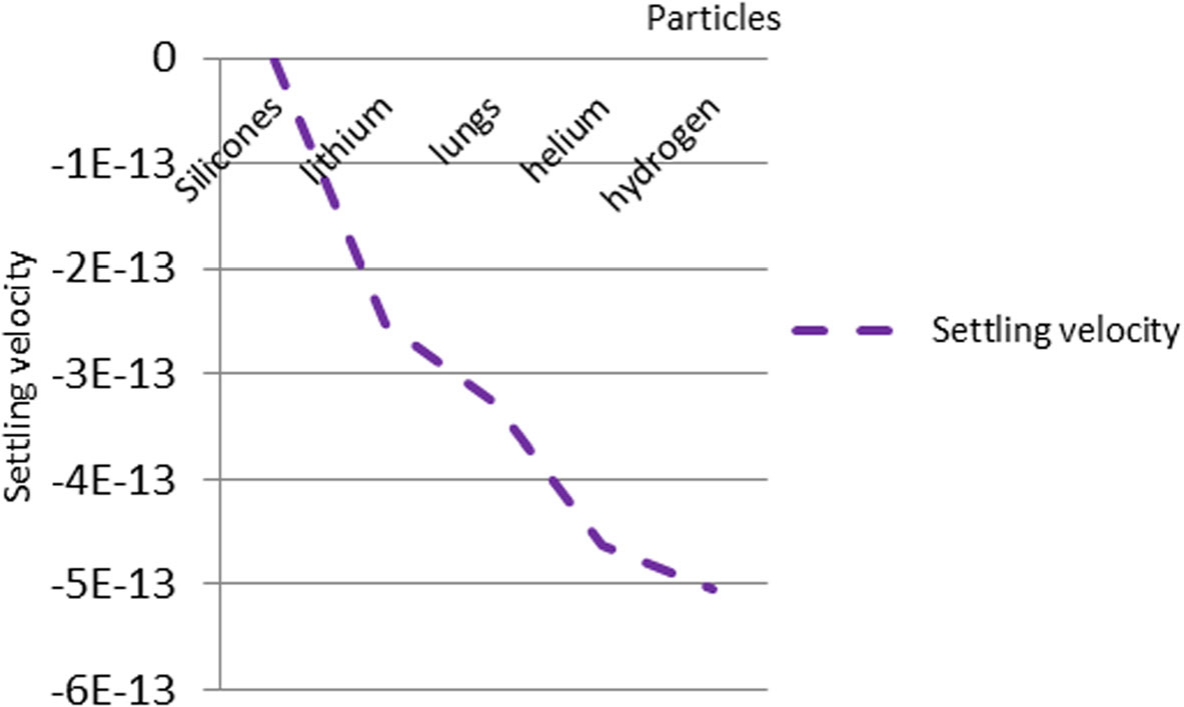
The settling velocity data for each particle

**Fig. 6:**
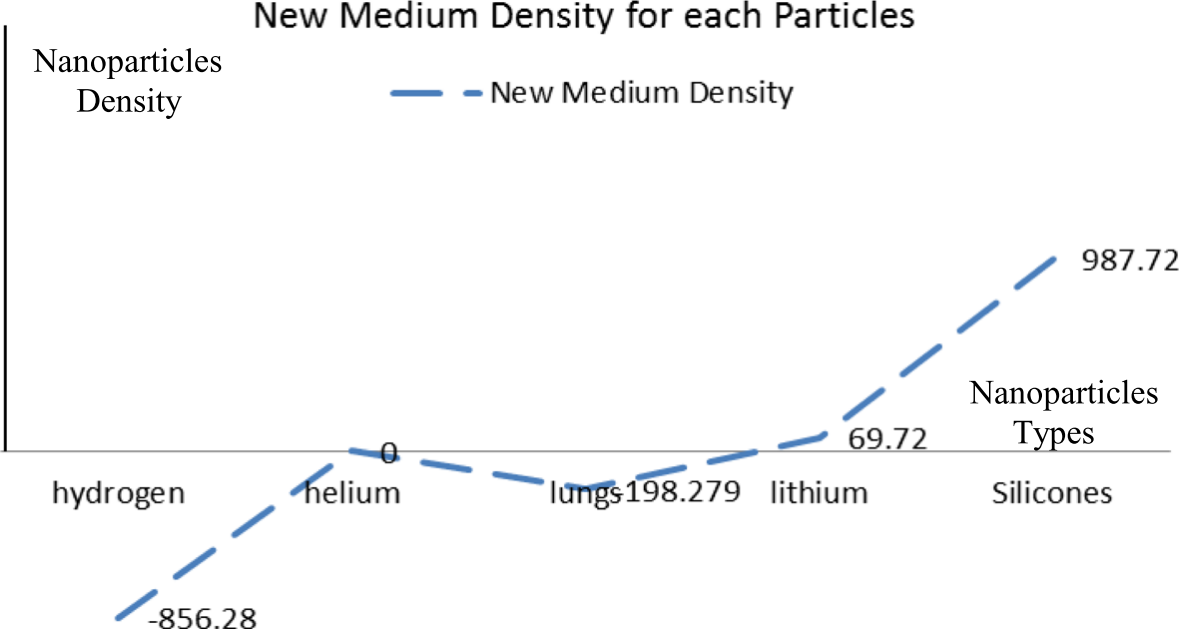
The effect of the settling velocity of nanoparticles on changing the lymphocyte density of cancer cells

In order to analyze the proposed design, the nanoparticles were applied to the following five types of materials: silicone, lithium, lung, helium, and hydrogen. The materials were chosen because of their low density. The lung nanoparticles were samples from nano-sized lung nodules. They appear encircling with white shadows in a chest X-ray or computerized tomography scan taken from the lung of the person and required to be undamaged. The proposed idea is based on the analytical model, which indicates that the smaller the density of nanoparticles, the smaller the dynamic viscosity will be. This will result in a decrease in fluid viscosity. It is shown that the types of materials and the density of each particle will affect settling velocity of nanoparticles at entry into the lymphatic fluid and the density of the lymphatic fluid. We considered the following parameters: acceleration of gravity (*g*) = 9.80665, particle diameter (*d*) = 10 A, initial density of lymph fluid (ρf) = 998.28, and dynamic viscosity = 0.0010 kg m^–1^ s^–1^ [24]. These parameters were selected by the assumption that the viscosity of the lymphatic fluid is very similar to the viscosity of the water and the very small difference does not affect the results of the model. Figure 4 illustrates the density of nanoparticles for five selected materials for application in the proposed analytical model. Figure 5 shows the settling velocity for each particle. Figure 6 shows the effect of the settling velocity of nanoparticles on altering the lymphocyte density of cancer cells. The results shown in Fig. 6 show that the settling velocity of the particles carries a negative value. This indicates that the nanoparticle after entering in the lymphatic fluid rapidly moves in the opposite direction toward their entry into the lymphatic fluid. In general, any object that moves in the negative direction has a negative velocity. This movement of the particle leads to reduced viscosity of the lymphatic fluid.

**Fig. 7:**
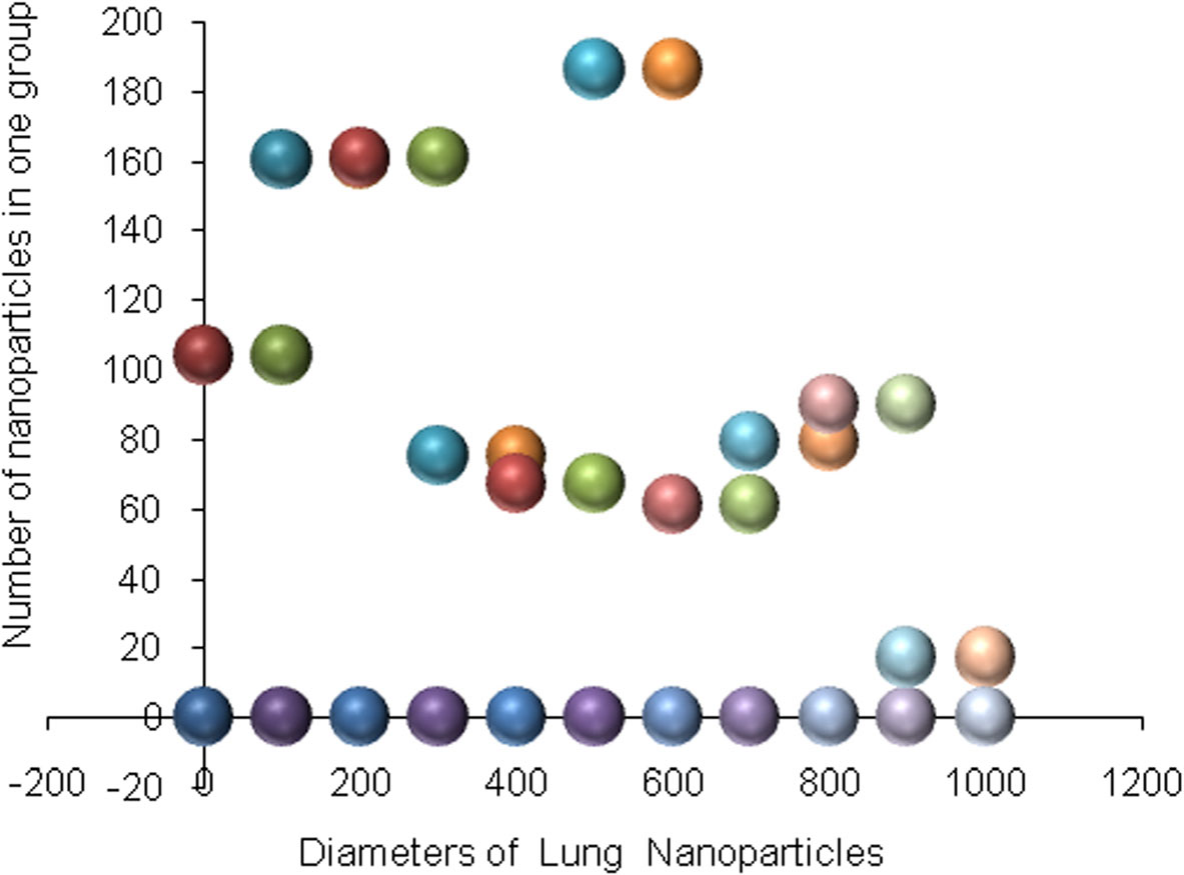
Group of nanoparticles in the lung cells and their number in one of the proposed groups

**Fig. 8:**
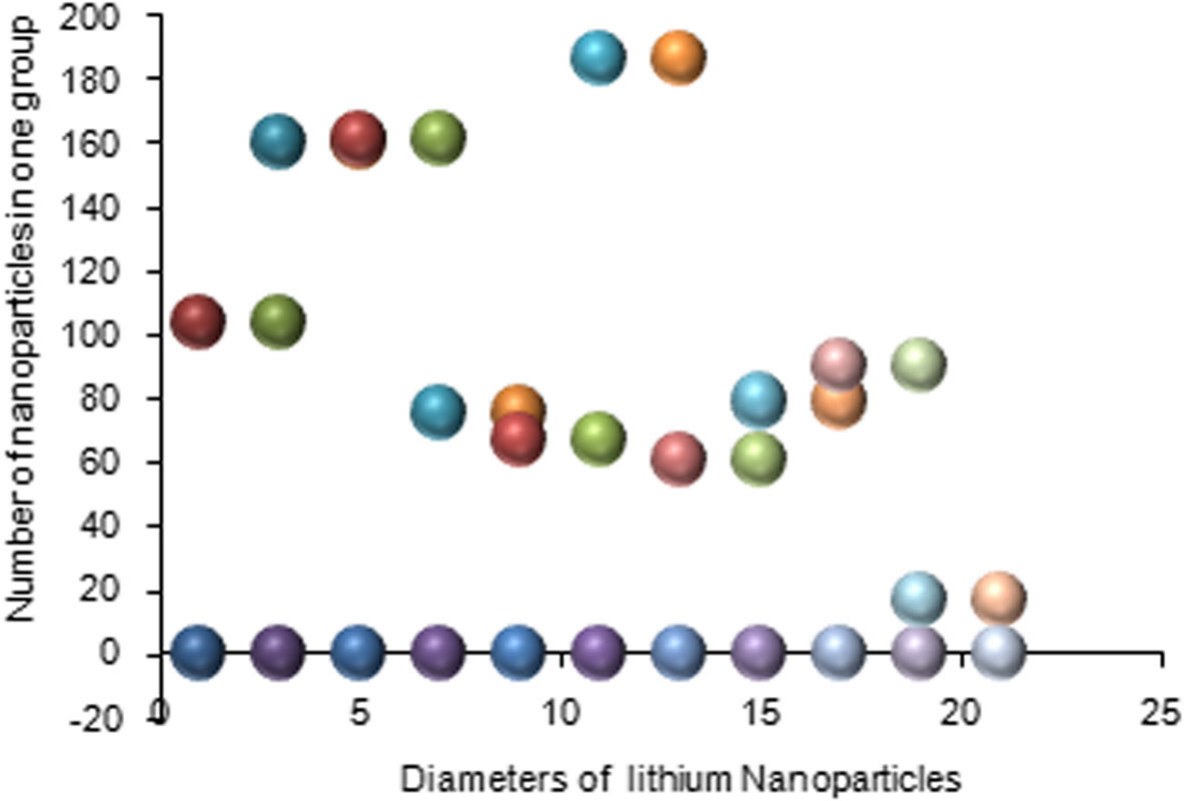
Group of nanoparticles in the lithium cells and their number in one of the proposed groups

**Fig. 9:**
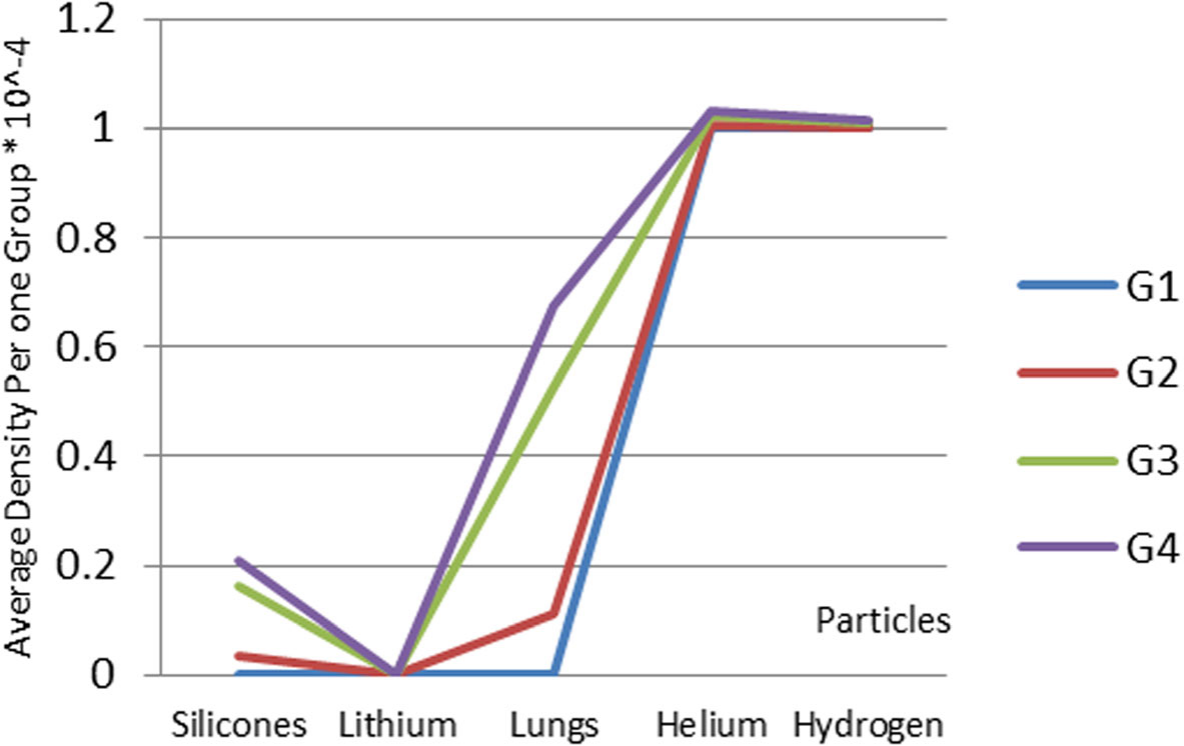
Different sets of materials proposed to have the mean highest density of both hydrogen and helium materials

**Fig. 10:**
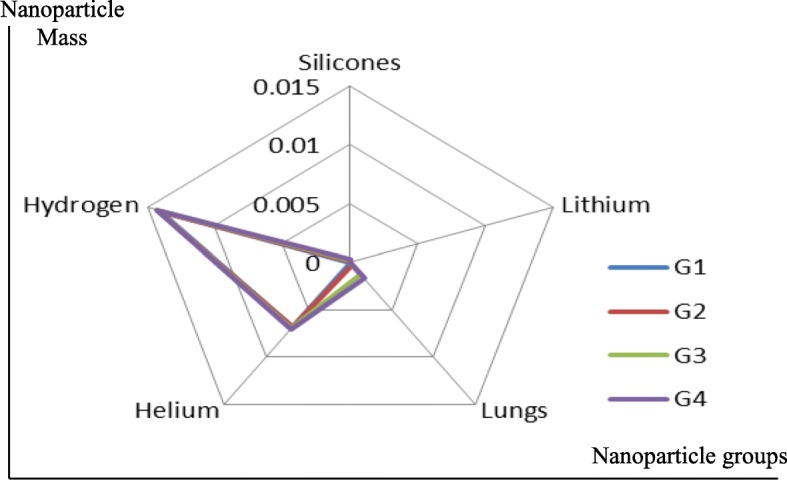
Average mass of a nanoparticle in a group

**Fig. 11:**
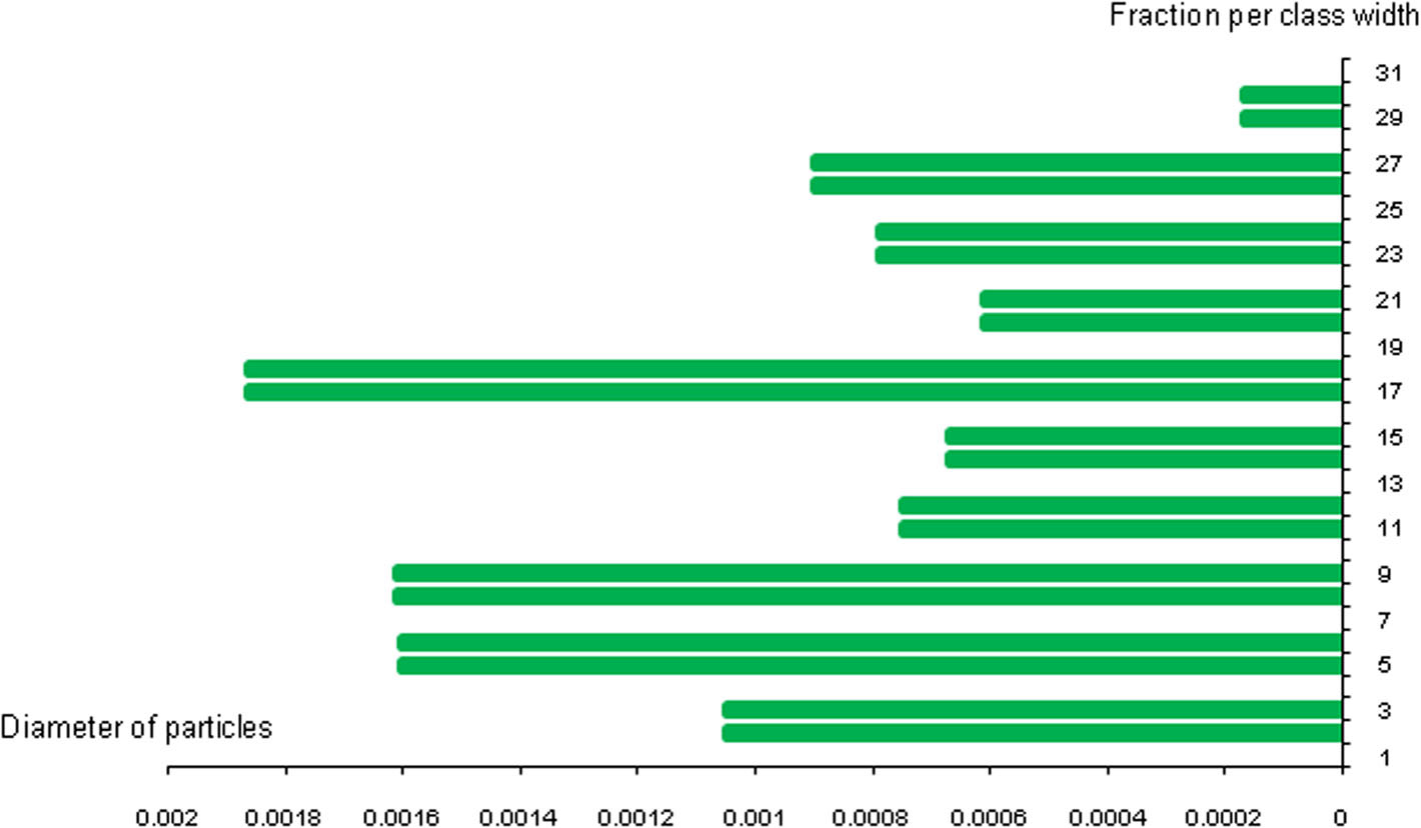
Diameters of the nanoparticles related to the group width

**Fig. 12:**
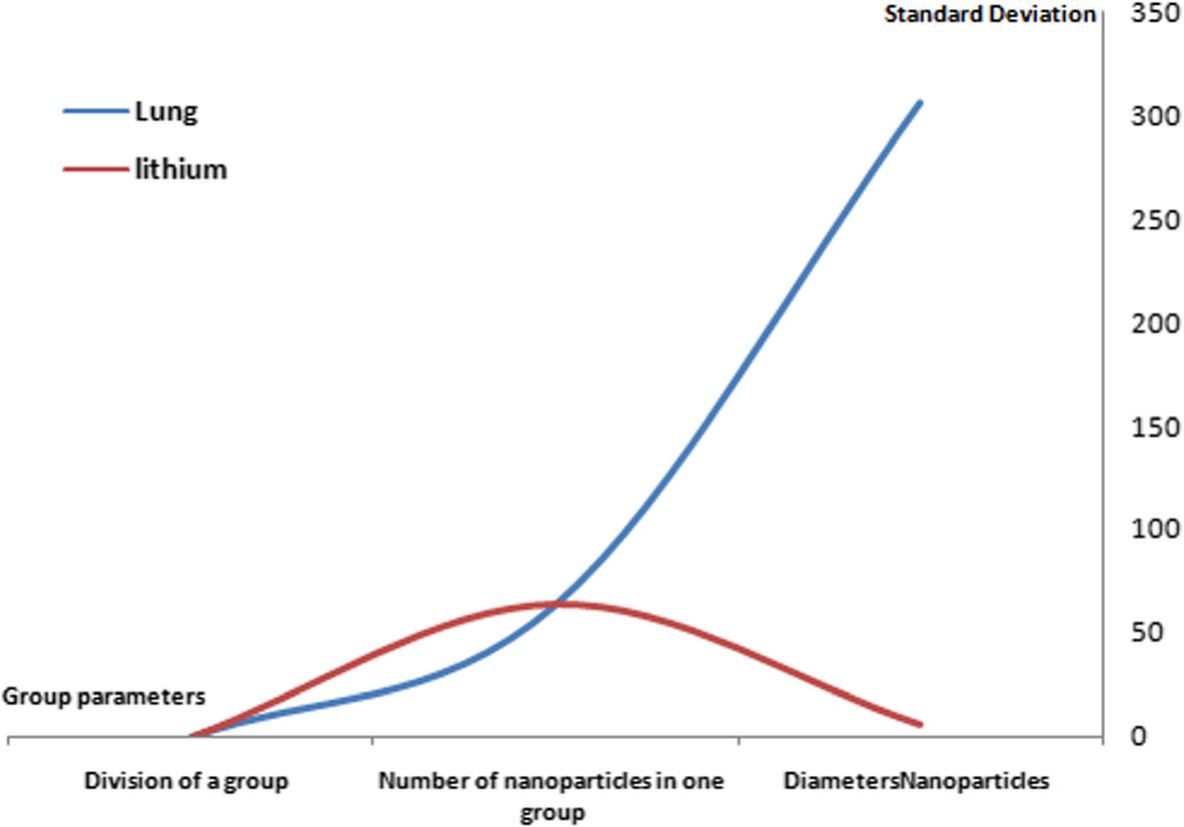
The standard deviation of lung and lithium nanoparticles coefficients

**Fig. 13:**
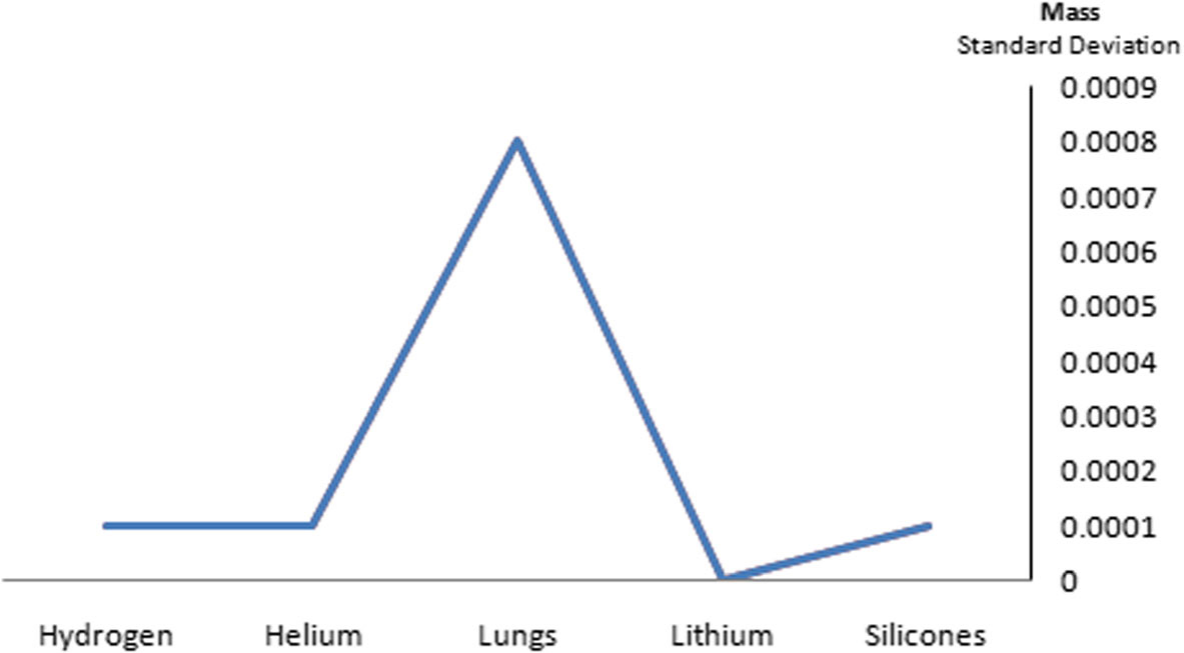
The standard deviation of the mass for particles of silicones, lithium, lungs, helium, and hydrogen in one group

Silicone nanoparticles showed the settling velocity of approximately − 2.87 × 10–15 m/s. This resulted in a decrease in viscosity of the lymphatic fluid to 987.72 kg/m^3^ for the initial density 998.28 kg/m^3^. The density is continuously reduced to a point where hydrogen produces extremely spectacular results, i.e., the complete collapse of lymphatic fluid resistance. The density of the lymphatic fluid − 856.28 kg/m^3^ with the negative sign indicated that there was no resistance from the lymphatic fluid to the flow of the nanoparticles, resulting in the complete collapse of the liquid fluid. Both the hydrogen and helium particles have a significant impact on the liquid viscosity due to the low density of the particles. Hence, it is important to use a drug system consisting of a group of nanoparticles for low-density materials. Figure 7 shows the relationship between the diameters of lung nanoparticles and the number of nanoparticles in one group. The figure shows that the higher the diameter of nanoparticles, the fewer their number in a group. This is clearly shown at the highest value of the nanoparticle diameter of 1000 nm, where the number of molecules in a group is 20 molecules. Figure 8 shows the relationship between the diameters of lithium nanoparticles and the number of nanoparticles in one group. This figure demonstrates the inverse relationship between the radius of nanoparticles and the number of molecules in a group where lithium particle diameters are significantly lower than the lung nanoparticles, where the number of nanoparticles in Fig. 7 is relatively low compared to the lithium particles as shown in Fig. 8. And the multicolor balls in both figures refer to different ranges of nanoparticle radii for each group, where each group contains a number of nanoparticles with different sizes. The best results can be obtained when hydrogen and helium particles are increased from other substances. A mixture of different materials should be used so that the properties of these substances can be used in the treatment process as well as to reduce viscosity. Figure 9 illustrates the different sets of materials proposed to have the mean highest density of both hydrogen and helium materials. Figure 10 shows the average mass of a nanoparticle in a group. It can be seen that the mass of both hydrogen and helium is the highest compared to the mass of particles of other substances. Figure 11 illustrates the relationship between the diameters of the nanoparticles and the width of its group or class. It is important to note that these results will open up a new area to reduce the resistance of the lymphatic fluid in tumors. This can be achieved using hydrogen nanoparticles of a size in the range of Angstrom. In addition to hydrogen nanoparticles, there may also exist a number of other substances in the same size. Figure 12 illustrates the standard deviation of a number of coefficients for both lung and lithium nanoparticles. These coefficients are limited to fractions of nanoparticles in a single group as well as their number in addition to the diameters of these nanoparticles. It is clear that the group fractions have the less value of the standard deviation. Hence, most of the fractions in the computational processes are around the mean of these values. Figure 13 shows the standard deviation of the mass for particles of silicones, lithium, lungs, helium, and hydrogen in one group. It is clear that the particles of the lung have the largest standard deviation and the lithium has the minimum value.

## Methods

The aim of this study was to establish a nano-drug delivery system capable of delivering the drugs effectively to the cancer cells. The following methodology was used to deliver nanoparticles:

i) Low-density nanoparticles

This study proposed the theoretical approach of nanoparticles as a low-density drug. This depends on the density and the settling velocity of the nanoparticles, as these nanoparticles can overcome the resistance of the lymphatic fluid.

ii) Preparation of anaerobic nanoparticles

This study uses the idea of nanoparticles possessing an antenna through which a connection can be made between nanoparticles and nano-controller. The transmission distance was assumed to be too small to match the composition of nanoparticles and also to fit the actual distance between them.

iii) Nano-controller design

Its function is to deliver the nanoparticle drug to cancer cells. Its role is to send signals to the nanoparticles and coordinate their actions and direct them to the lymphatic fluid of tumors.

iv) Searching for the target lymphatic nodes

The lymphatic nodes are searched using compressive binary search algorithm. This algorithm is characterized by high-speed search, which makes nanoparticles more accessible to infected cells than the conventional methods. The primary supervisor behind the performance of the nanoparticles is the nano-controller. It directs nanoparticles to the infected cells by following this algorithm to ensure that an appropriate number of molecules are in proportional density to the lymphatic fluid.

## Conclusion

There have been various studies managing the treatment of malignant growth utilizing nanoparticles. The lymphatic liquid in tumors plays a substantial role in the obstruction of medication to the cancer cells. We developed an intelligent drug delivery system containing a consortium of nanoparticles. The proposed design demonstrates that small nanoparticles result in low density of the fluid. The results indicated that hydrogen particles are most efficient in reducing resistance toward lymphatic liquid owing to their smaller size. Furthermore, the design involves an anaerobic nano-controller that can determine the state and area of the particles. This technique conveys the medication to the infected cell more effectively.

## Data Availability

The datasets supporting the results of this article are included within the article.

## Abbreviations

LN: Lipid nanoparticles
NLC: Nanostructured lipid carriers
PN: Polymeric nanoparticles
SLNs: Solid lipid nanoparticles
